# Qingfeiyin Decoction Inhibits H1N1 Virus Infection *via* Modulation of Gut Microbiota and Inflammatory Pathways in a Murine Model

**DOI:** 10.3389/fphar.2022.874068

**Published:** 2022-05-23

**Authors:** Xianping Li, Mingzhe Wang, Chang Liu, Yuchun Xiao, Mengde Li, Chengjun Ban, Yuanming Huang, Miao Cheng, Liqiong Song, Guoxing Liu, Shan Lu, Chengxiang Wang, Zhihong Ren

**Affiliations:** ^1^ State Key Laboratory of Infectious Disease Prevention and Control, National Institute for Communicable Disease Control and Prevention, Chinese Center for Disease Control and Prevention, Research Units of Discovery of Unknown Bacteria and Function (2018RU010), Chinese Academy of Medical Sciences, Beijing, China; ^2^ Respiratory Department, Dongzhimen Hospital Affiliated to Beijing University of Chinese Medicine (BUCM), Beijing, China; ^3^ Department of Internal Medicine, Gulou Hospital of Traditional Chinese Medicine of Beijing, Beijing, China; ^4^ School of Computer Science and Information Engineering, Hefei University of Technology, Hefei, China; ^5^ Beijing University of Chinese Medicine Third Affiliated Hospital, Beijing, China; ^6^ Linwei Liu Zunji Clinic of Traditional Chinese Medicine, Weinan, China

**Keywords:** Qingfeiyin decoction, traditional Chinese medicine, H1N1 virus, gut microbiota, transcriptome, inflammatory pathways

## Abstract

Influenza virus-caused lung infection and its pandemic outbreaks are a persistent public health challenge. The H1N1 subtype is the most common type of influenza infection observed in humans. Maxingshigantang decoction, a classic formula of Chinese herbal medicine, has been used for the prevention and treatment of respiratory infection for many centuries. Qingfeiyin decoction, based on Maxingshigantang, has been used in the clinic for decades. To explore the underlying mechanisms, according to the traditional Chinese medicine theory “the lung and the large intestine are interior–exterior,” which can be translated to the “gut–lung axis” in a contemporary term, the composition of gut microbiota was determined using 16S rRNA and the transcriptome of the colon was determined by RNA sequencing. The results showed that Qingfeiyin decoction decreased the viral load, alleviated the lung injury, increased the survival rate, partly restored the shortening of the colon caused by the H1N1 virus, and downregulated inflammatory pathways including MAPK, TNFα, and JAK-STAT signaling pathways. Qingfeiyin decoction increased the relative abundance of the genera of *Coprococcus* *, Ruminococcus, Lactobacillus*, and *Prevotella* and prevented the H1N1 virus-induced decrease in the abundance of the genera of *Escherichia*, *Parabacteroides*, *Butyricimonas,* and *Anacrotruncus.* These results will help better understand the mechanisms for Qingfeiyin decoction’s protective effect against influenza virus infection.

## Introduction

Coronavirus disease (COVID-19) caused by severe acute respiratory syndrome coronavirus 2 (SARS-CoV-2) has drawn great attention worldwide since COVID-19 was first reported in December 2019 (Zhou et al., 2020; Zhu et al., 2020). However, the global threat of influenza, especially the H1N1 influenza A infection, cannot be neglected. The Spanish flu resulted in approximately 500 million infected cases and 50 to 100 million deaths worldwide (Trilla et al., 2008). Mexico flu also infected over 200,000 people and caused 2,000 deaths in 2009 (Franco-Paredes et al., 2009). Therefore, influenza remains a major burden to public health due to the morbidity and mortality it causes. Vaccine is an effective strategy to prevent influenza; however, current influenza vaccines could not provide sufficient protection against the potential influenza pandemic because of virus evolution and rapid mutation of the viral epitopes targeted by the vaccine (Nachbagauer and Palese, 2020).

Traditional Chinese medicine (TCM) plays an important role in treating viral pneumonia caused by SARS and SARS-CoV-2 as it has proved efficacy and fewer side effects (Shao et al., 2020; Xi et al., 2020). Maxingshigantang decoction, a classic prescription for treating lung diseases, has been used to prevent and treat respiratory infections and fevers for many centuries (Hsieh et al., 2012). Maxingshigantang decoction, composed of four kinds of herbs, includes *Ephedra sinica* Stapf, *Prunus armeniaca L*., *Lycopodiella cernua (L.)* Pic. Serm., and *Glycyrrhiza* glabra *L.* Qingfeiyin decoction (QFYD), which is formulated based on Maxingshigantang decoction, is comprised of 12 herbs. QFYD has been prescribed by experienced clinicians at Dongzhimen Hospital, Beijing University of Chinese Medicine in the clinic for decades. Multi-compounds of QFYD may target different aspects of the influenza virus which ensures its efficiency to combat the influenza virus and its mutants.

Growing evidence indicates that influenza infection alters the composition of gut microbiota in H1N1 influenza patients and in mice infected with the H1N1 virus (Chen et al., 2019; Gu et al., 2020). TCM can interact with the gut microbiota in many ways. On the one hand, TCM can modulate the composition and metabolism of gut microbiota, and on the other hand, gut microbiota can also affect and participate in the metabolism of TCM (Feng et al., 2019). In addition, “the lung and the large intestine are interior-exterior” of TCM theory indicates that lung diseases are closely related to large intestine function, which well coincides with the current concept “gut-lung axis.” Although the QFYD has been used in the clinic for decades, the underlying mechanism, in particular, whether QFYD protects against influenza infection through modulating gut function is unclear. In the current study, we focused on investigating the effect of QFYD on the gut microbiota and colon transcriptome. The data showed that influenza infection shortened the length of the colon and altered the composition of gut microbiota, including an increased relative abundance of the Proteobacteria phylum and a decreased Firmicutes phylum. QFYD reversed these alterations caused by influenza, as indicated by the longer colon length, restoration of gut microbiota, and downregulation of inflammatory pathways in the colon.

## Materials and Methods

### Ethics Statement

This study was approved by the Ethics Review Committee of the National Institute for Communicable Disease Control and Prevention at the Chinese Center for Disease Control and Prevention (Beijing, China).

### H1N1 Influenza Virus Culture

Madin–Darby canine kidney cells were cultured in DMEM containing 10% fetal bovine serum, 0.1 mg/ml streptomycin, and 100 U/ml penicillin under a 4.5% CO_2_ incubator at 35°C. The influenza virus A/PR/8/34(H1N1) was obtained from the National Institute for Viral Disease Control and Prevention of China and was propagated in Madin–Darby canine kidney cells.

### Qingfeiyin Decoction Formulation and Identification

One prescription of QFYD consisted of 12 herbs including Ephedra sinica Stapf (Mahuang, fried, 6 g), *Prunus armeniaca L.* (Kuxingren, fried, 6 g), *Lycopodiella cernua (L.)* Pic. Serm. (Shigao, 30 g), *Scutellaria baicalensis* Georgi (Huangqin, 15 g), *Bombyx* batryticatus (Jiangca, 10 g), Reynoutria japonica Houtt. (Huzhang, 15 g), *Scleromitrion* diffusum (Willd.) R.J.Wang (Baihuaseshecao, 15 g), *Houttuynia cordata* Thunb. (Yuxingcao, 30 g), *Trichosanthes kirilowii* Maxim (Quanguolou, 30 g), *Platycodon grandiflorus* (Jacq.) A. DC. (Jiegeng, 10 g), *Forsythia suspensa* (Thunb.) Vahl (Lianqiao, 30 g), and *Glycyrrhiza glabra* L. (Gancao, 6 g). Qingfeiyin granulate was purchased from Beijing Tcmages Pharmaceutical Co., Ltd.

The QFYD granules made from its concentrated water extract were analyzed with the UPLC-MS platform by Biomsomics Biotechnology Co., Ltd. The compounds were separated using a Waters Acquity™ UPLC system with a column temperature of 45°C. The autosampler was maintained at 4°C and the flow rate of the mobile phase was 2 μl/min. The mobile phase consisted of the A phase (water + 0.1% formic acid) and the B phase (acetonitrile + 0.1% formic acid) using a gradient system. To detect the peak intensity, mass spectrometry was conducted in the negative and positive modes under the following conditions: N2 flow of 20 arb, source temperature of 150°C, ion spray voltage (IS) of 2.3 kV, and a scan time of 25 min. The data were analyzed by Peakview software, the parameters were set as follows: mass error <5 ppm, isotope ratio difference <10; library score >70 (Huang et al., 2020).

### Influenza Infection Mouse Model and Intervention by Qingfeiyin Decoction

Female C57BL/6J mice (5 weeks, 14–15 g) were purchased from Beijing Vital River Laboratory Animal Technology Co., Ltd. (Beijing, China). All animal experiments were performed in accordance with protocols approved by the Welfare and Ethical Inspection in Animal Experimentation Committee at the Chinese CDC. All mice were housed in groups of four per cage in the China CDC Animal Center and were allowed free access to water and food under a 12 h light cycle. After 7 days of adaption, 32 mice were divided randomly into four groups (*n* = 8 per group): control, PBS, oseltamivir, and QFYD group. Another 32 mice were also randomly divided into four groups for the survival test. Except for the control mice, all the other mice were infected with 360 PFU H1N1 influenza viruses by nasal administration, and 720 PFU was administrated for the survival test. QFYD granules were administrated once a day by gavage at the dose of 1.54 g/(kg∙d) in 300 μl PBS to the mice of the QFYD group after 24 h of H1N1 infection. Oseltamivir, at the dose of 28.46 mg/(kg∙d) in 300 μl PBS, and 300 μl PBS was administrated to mice in the oseltamivir group and PBS group, respectively. After 7 days of treatment, the lung and colon tissues and cecum content were collected for the analyses as described in the followings, and the mice in the survival experiment were observed daily through the period of 14 days.

### H1N1 Virus Loading Detection

The load of H1N1 influenza virus in the lung was determined using influenza A virus nucleic acid test kit (Jiangsu Hechuang Biotechnology Co., Ltd.) according to manufacturer’s instructions. Briefly, the total mRNA was extracted from lung tissue, and a 20 μl reaction system containing 2 μl RNA, 17.2 μl PCR reaction mixture, and 0.8 μl enzyme reaction mixture was performed in an ABI 7500 Real-Time PCR System (ABI, United States). The reaction conditions were as the follows: 42°C 10 min, 94°C 10 s, followed by five cycles of 94°C for 5 s, 50°C for 20 s, and 72°C for 20 s, then by 40 cycles of 94°C for 5 s, 56°C for 50 s, and 72°C for 15 s.

### Histopathological Analysis

Lung and colon tissues were collected and fixed in 10% formalin solution for 24 h and were then embedded in paraffin and sliced into 5 μm thick sections, which were stained with hematoxylin and eosin (H/E). The H/E sections were observed under a light microscope by two experienced pathologists.

### 16S rRNA Sequencing and Data Analysis

Total genome DNAs of 18 cecum content of mice were extracted according to the manufacturer’s protocols. DNA concentration was determined using the Equalbit dsDNA HS Assay Kit, and the integrity of DNA was measured with 1% agarose. V3 and V4 hypervariable regions of 16S rRNA were selected and amplified using primer pairs: forward primer (5′-CCTACGGGNGGCWGCAG-3′) and reverse primer (5′-GACTACHVGGGTATCTAATCC-3′), and the amplicons and quality control of the raw data were conducted on the Illumina Hiseq 2,500 platform by Beijing Genomics Institute Co., Ltd. (Beijing, China). Qualified purified chimeric sequences were grouped into OTUs (operational taxonomic units) clustering by USEARCH (v7.0.1090) at a 97% threshold. Based on OTU analysis results, Ace, Chao, and Shannon indexes were calculated using the method of random sampling. The PCA (principal components analysis) was based on the OTU abundance using the unweighted unifrac analysis. Histograms were generated using the software R (v3.1.1) at the phylum and genus taxonomic levels. Linear discriminant effect size (LEfSe) analysis (http://huttenhower.sph.harvard.edu/galaxy/) was conducted online using nonparametric factorial Kruskal–Wallis and Wilcoxon rank-sum tests. The threshold was set at 2.0 for discriminative features (Segata et al., 2011). The original data can be downloaded from the NCBI SRA database (accession number: PRJNA765210, SRP 338305).

### Transcriptome Analysis of the Colon and Quantitative Real-Time PCR

Total RNA of colon tissue was extracted using the Trizol agent according to the manufacturer’s instructions. After the detection of concentration and integrity by Fragment Analyzer, the qualified RNA sample was used to construct a data library and sequenced on the BGISEQ-500 platform by Beijing Genomics Institute Co., Ltd. The clean data obtained from raw data by removing adapter-containing and more than 10% of N-containing reads were aligned to the reference gene using Bowtie2 software and the reference genome using the HISAT software (Kim et al., 2015). The matched reads were normalized to fragments per kilobase million (FPKM) by RSEM software for gene expression analysis (Li and Dewey, 2011). Gene set enrichment analysis (GSEA) was analyzed by GSEA software (version 3.0), with a nperm of 1,000, min 15, max 500. The original data can be downloaded from the NCBI SRA database (accession number: PRJNA765456, SRP 338360).

The expression levels of *MUC2*, *Cyp2c55*, *Nupr1*, and *Slfn4* were validated by quantitative real-time PCR (RTq-PCR). The sequences of primers are listed in Table 1. Total RNA of colon tissue was extracted and reverse-transcribed to cDNA according to the manufacturer’s instructions. A total of 20 μl of reaction, containing 2 μl cDNA, 10 μl TB Green Premix Ex TaqTM II, 300 nM primer, and 0.4 μl ROX Reference Dye Ⅱ, was performed in an ABI 7500 Real-Time PCR system under the following conditions: 40 cycles at 95°C for 30 s, 95°C for 3 s, and 60°C for 30 s, followed by dissolution curve analysis after each cycle at 95°C for 15 s and 60°C for 60 s. The folds were calculated by the comparative 2^−ΔΔCt^ method, and β-actin was used as an internal control.

**TABLE 1:** Sequences of real-time PCR primers.

Gene name	Sequence
*β-Actin-F*	TGG​ATG​ACG​ATA​TCG​CTG​CG
*β-Actin-R*	AGG​GTC​AGG​ATA​CCT​CTC​TT
*MUC2-F*	GCT​GAC​GAG​TGG​TTG​GTG​AAT​G
*MUC2-R*	GAT​GAG​GTG​GCA​GAC​AGG​AGA​C
*Cyp2c55 F*	AAT​GAT​CTG​GGG​GTG​ATT​TTC​AG
*Cyp2c55 R*	GCG​ATC​CTC​GAT​GCT​CCT​C
*Nupr1 F*	CCC​TTC​CCA​GCA​ACC​TCT​AAA
*Nupr1 R*	TCT​TGG​TCC​GAC​CTT​TCC​GA
*Slfn4 F*	GGC​TCC​CTG​CGT​AAA​GGA​AC
*Slfn4 R*	GGG​TAA​CAT​ATT​TTC​GCG​CTT​GA

### Statistical Analysis

Statistical analysis was performed with GraphPad Prism (v.5.0). Comparisons of bodyweight, lung index, viral loading, the length of the colon, α diversity, and expressions of *MUC2*, *Cyp2c55*, *Nupr1*, and *Slfn4n* were analyzed using one-way analysis of variance (ANOVA). The survival rate was analyzed by the Log-rank (Mantel-Cox) test. PCOA was analyzed using a nonparametric MANOVA based on Adonis. *p <* 0.05 was considered statistically significant.

## Results

### The Major Chemical Components of Qingfeiyin Decoction

A total of 73 chemical components were identified in the QFYD granules by UPLC-QTOF-MS, and the intensity of over 1000,000 was selected and listed in Table 2. Among the top 16 chemical components, baicalein intensity was the highest, reaching 14,003,576 in the positive ion model, and forsythoside I was the highest, reaching 6,289,474 in the negative ion model. Six of the 16 chemical components were flavonoids (Table 2).

**TABLE 2:** The chemical components with relative high abundance in QFYD detected by UPLC-MS.

Index	Name	Formula	Adduct	Intensity	Category
16	Baicalein	C15H10O5	+H	14003576	Flavonoids
34	Forsythoside I	C29H36O15	-H	6289474	Phenylethanoid glycosides
45	Wogonoside	C22H20O11	-H	3417720	Flavonoids
58	Chrysosplenetin B	C19H18O8	-H	3297123	Flavonoids
42	Forsythin	C27H34O11.HCOOH	-H	2519910	Lignans
48	Glycyrrhizic acid	C42H62O16	-H	1938992	Triterpenoids
1	L(+)-arginine	C6H14N4O2	+H	1658039	Amino acids
38	Pinoresinol-glucoside	C26H32O11	-H	1422811	Lignans
20	Citric acid	C6H8O7	-H	1379763	Organic acids
19	Sorbitol	C6H14O6	-H	1332137	Polyols
57	Chrysin	C15H10O4	-H	1297553	Flavonoids
37	Liquiritin	C21H22O9	-H	1289208	Flavonoids
64	Glabridin	C20H20O4	-H	1211750	Flavonoids
4	Stachydrine	C7H13NO2	+H	1153648	Alkaloid
35	Calceorioside B	C23H26O11	-H	1091145	Phenylethanoid glycosides
63	Sanggenon H	C20H18O6	-H	1033756	Flavonoids

### The Effect of Qingfeiyin Decoction on Lung Damage in Mice Infected by H1N1 Influenza Virus

**FIGURE 1:**
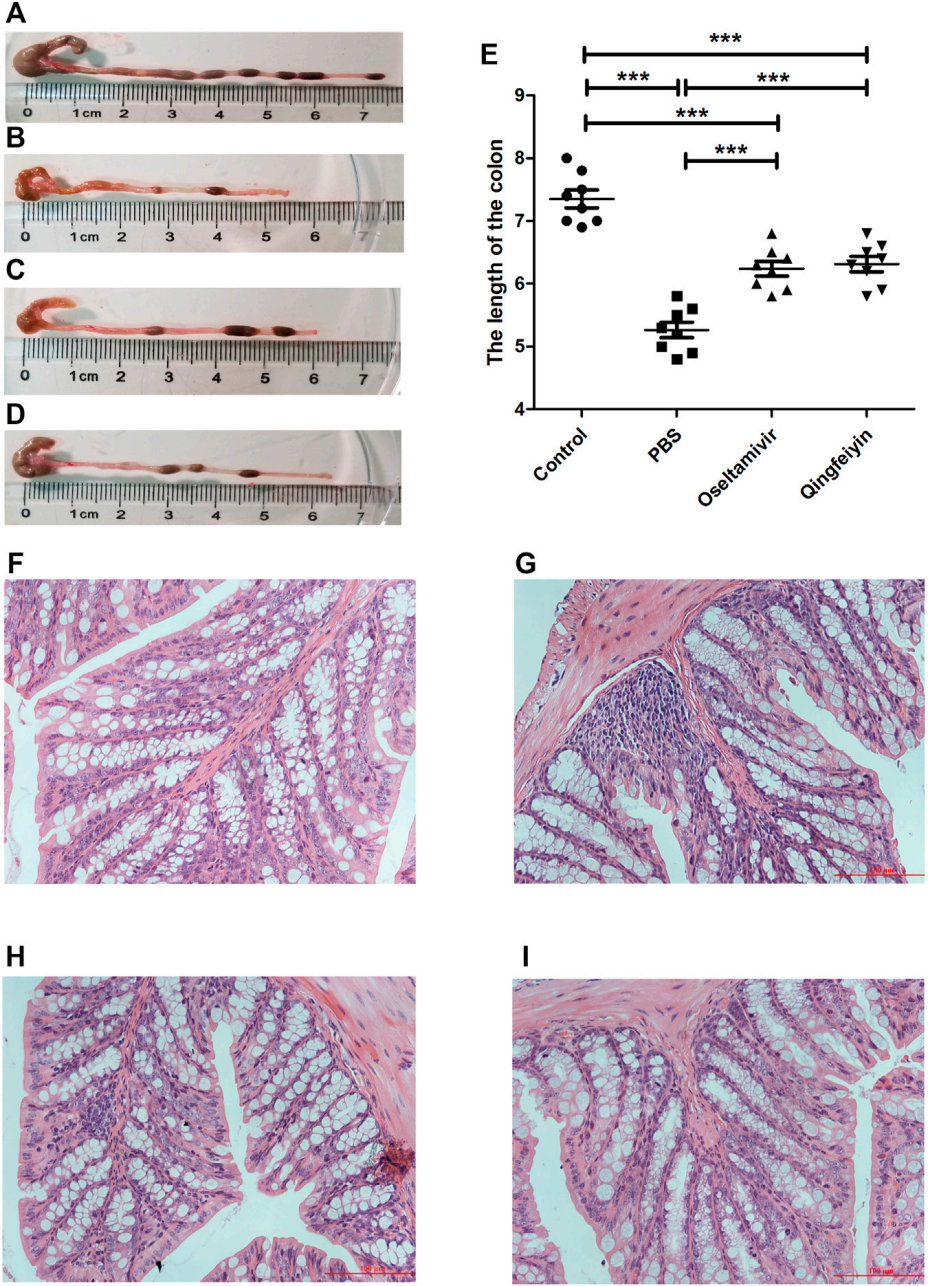
The effect of QFYD on bodyweight, viral load, lung index, survival rate, and lung tissue. QFYD increased the bodyweight of mice **(A)**, decreased the viral load in the lung **(B)**, lung index **(C)**, and enhanced the survival rate **(D)** after 7 days of treatment with QFYD in mice infected with the H1N1 virus. **(E–H)** is representative H/E staining pictures of control, PBS, oseltamivir, and QFYD group (x 200), respectively. The data of bodyweight, lung index, and viral load were analyzed by analysis of variance (ANOVA), and the survival rate was analyzed by the Log-rank (Mantel-Cox) test. *p* < 0.05 means the statistical difference. *p < 0.05; **p < 0.01; ***p < 0.001.

Compared to the bodyweight (BW) of the normal mice (16.79 ± 0.66 g), the mice infected with the H1N1 virus had lower BW (13.44 ± 0.54 g) on day 7 post-infection. The treatment with oseltamivir or QFYD prevented the infection-induced weight loss (15.17 ± 0.89 g and 15.36 ± 0.95 g, respectively) (Figure 1A). On day 3 post-infection, the viral load in the lung was 6.17 ± 0.26 in the PBS group, 4.90 ± 0.32 in the oseltamivir group, and 4.51 ± 0.47 in the QFYD group (Figure 1C). The lung index (lung weight/bodyweight) of normal mice was 0.59 ± 0.04, and virus infection increased the lung index compared to normal mice. The lung indices in both the oseltamivir and QFYD groups were significantly lower than those in the PBS group after 7 days of treatment (Figure 1B), suggesting the protective role of Oseltamivir and QFYD treatment.

Half of the mice infected with 720 PFU died on day 6 post-infection, and all mice died on day 8 post-infection in the PBS group. However, the oseltamivir and QFYD groups had delayed occurrence of mortality to day 7 post-infection, and the analysis of the Log-rank (Mantel-Cox) test showed that the survival rate of oseltamivir or QFYD groups was significantly higher than that in the PBS group, and there was no significant difference in survival rate between oseltamivir and QFYD (Figure 1D). The H/E staining picture seen in Figures 1E–H showed severe infiltration of inflammatory cells, significant thickness of the alveolar wall, and partly consolidated pulmonary alveolus in the PBS-treated infection mice compared to the uninfected control group. After 7 days of treatment with oseltamivir or QFYD, the areas of pulmonary consolidation and the number of infiltrating inflammatory cells were smaller, and the alveolar wall became thinner compared to the PBS group (Figures 1E–H).

### The Effect of Qingfeiyin Decoction on the Colon of Mice Infected by H1N1 Influenza Virus

**FIGURE 2:**
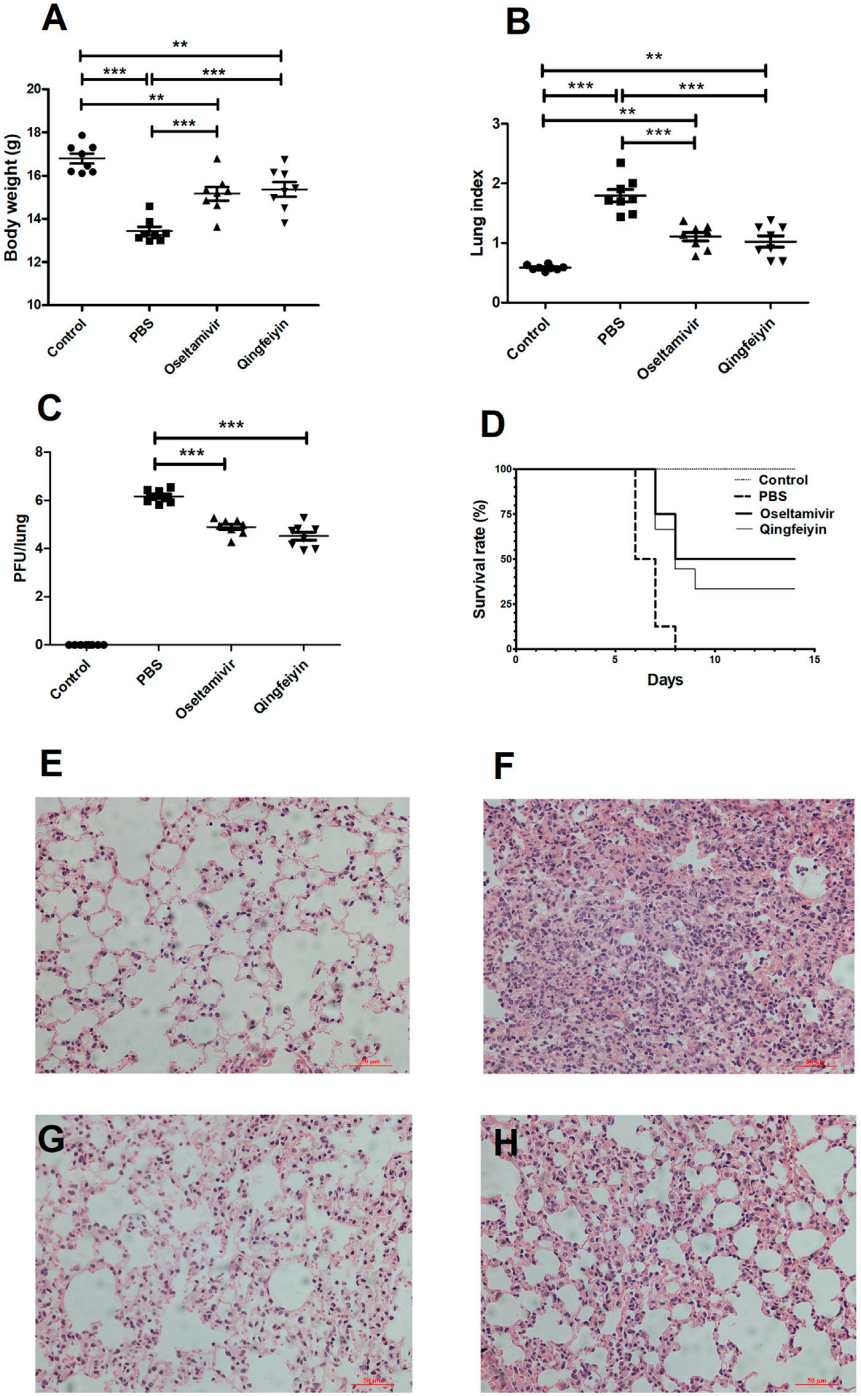
The effect of QFYD on the colon of mice infected with the H1N1 virus. Compared to the colon of normal mice **(A)**, the H1N1 virus shortened the length of the colon **(B)**, oseltamivir **(C)**, and QFYD **(D)** restored the length of the colon to a certain extent. **(E)** was the comparison of the colon length of four groups. **(F–I)** is representative H/E staining pictures of control, PBS, oseltamivir, and QFYD group (x 200), respectively. The data of colon length was analyzed by analysis of variance (ANOVA). *p* < 0.05 means the statistical difference. ***p < 0.001.

The colon length of infected PBS group mice was significantly shorter compared to the uninfected control mice on day 7 post-infection (Figures 2A, B). Both Oseltamivir and QFYD treatments restored the length of the colon and had significantly longer colon length compared with the PBS group (Figures 2C, D), and there was no significant difference between oseltamivir and QFYD treatment (Figures 2C–E). H/E staining of the colon showed larger infiltrating masses of inflammatory cells, reaching 0–2 in each visual field (Figure 2G). Smaller inflammatory cell masses were rarely found in the oseltamivir and QFYD groups (Figures 2H, I). No inflammatory cell mass was found in the uninfected control mice (Figure 2F).

### The Effect of Qingfeiyin Decoction on the Transcriptome of Mice Colon

**FIGURE 3:**
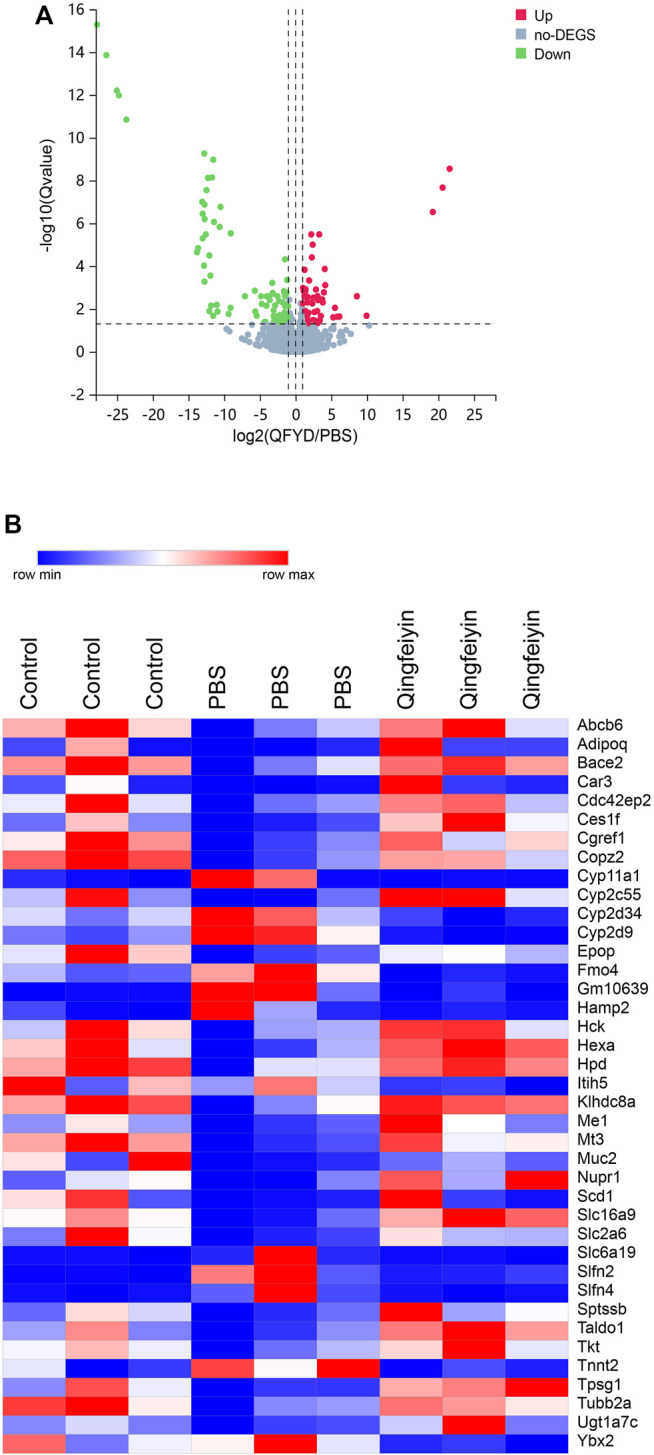
The overview of lung transcriptome of colon tissue. **(A)**, a volcano plot of up and downregulation genes. **(B)**, a heatmap of differential genes.

To explore the effect of QFYD on gene expression of the colon, RNA sequencing was performed. Compared to the PBS group, there were 149 differentially expressed genes in the QFYD group, including 49 upregulated genes and 100 downregulated genes. The volcano plot indicated the downregulated genes had an advantage over upregulated genes (Figure 3A).

The heatmap showed 39 differential genes with higher relative abundance, which were selected from 149 differential genes. The relative abundances of *Muc2*, *Cyp2c55*, *Nupr1*, and *Ces1f* genes were remarkedly higher, and *Slfn4*, *Cyp2d9*, and *Slfn2* were lower in the QFYD group compared to those of the PBS group (Figure 3B).

**FIGURE 4:**
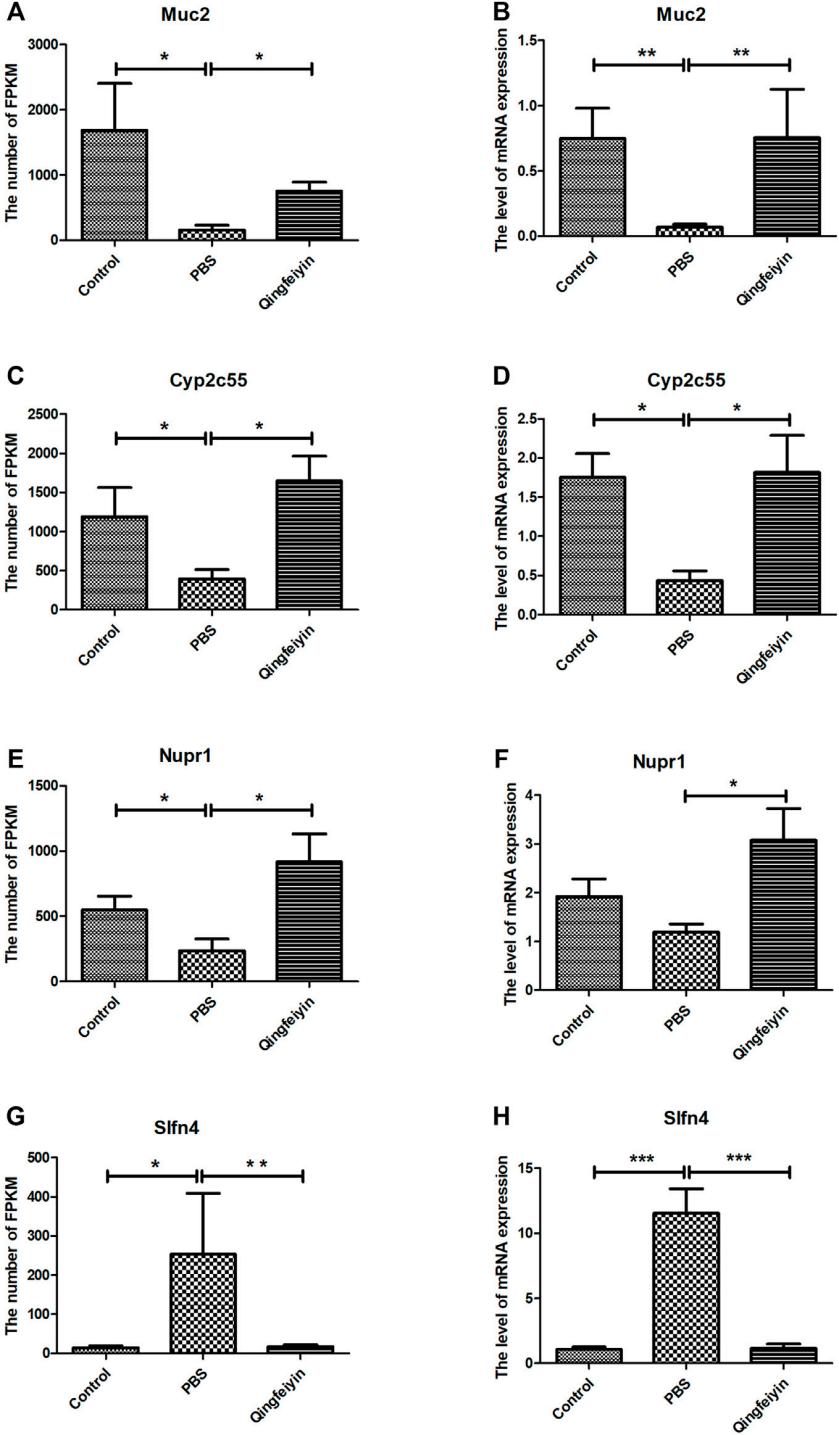
The expression and verification of the key differential genes. **(A,C,E,G)** is the expression of *Muc2*, *Cyp2c55*, *Nupr1*, and *Slfn4* in three groups by RNA seq, respectively. **(B,D,F,H)** are the relative abundances of mRNA expression of *Muc2*, *Cyp2c55*, *Nupr1*, and *Slfn4* by QRT-PCR. The data were analyzed by ANOVA. *p* < 0.05 means the significant differences. *p < 0.05; **p < 0.01; ***p < 0.001.

The FPKMs of *Muc2*, *Cyp2c55*, *Nupr1*, and S*lfn4* by RNA sequence were shown in Figures 4A, C, E, G. In the meantime, some downregulated genes (*Muc2*, *Cyp2c55*, and *Nupr1*) and upregulated gene (*Slfn4*) in response to H1N1 virus infection in mice were verified by Q-RTPCR (Figures 4B, D, F, H). The trends of upregulation or downregulation of four genes were consistent between FPKM and relative abundance, indicating that the results based on FPKM were reliable.

**FIGURE 5:**
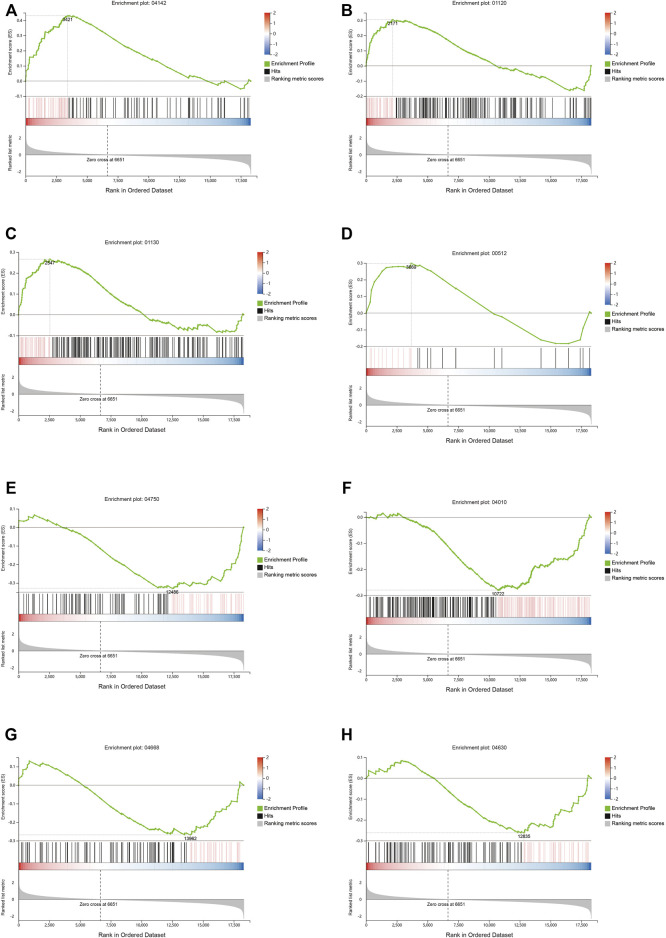
Signaling pathway analysis by Gene Set Enrichment Analysis (GSEA). Upregulated pathways included lysosome **(A)**, microbial metabolism in diverse environments **(B)**, biosynthesis of antibiotics **(C)**, and mucin type o-glycan biosynthesis **(D)**, Downregulated pathways included inflammatory mediator regulation of TRP channels **(E)**, MAPK signaling pathway **(F)**, TNFα signaling pathway **(G)**, and Jak-STAT signaling pathway **(H)**.

The differential gene analysis can determine the pathways affected by QFYD, but it cannot determine the direction, i.e., up- and downregulated. Therefore, a GSEA analysis was performed. GSEA showed that QFYD upregulated several pathways including lysosome, microbial metabolism in diverse environments, biosynthesis of antibiotics, and mucin type O-glycan biosynthesis pathway (Figures 5A–D). The upregulation of mucin type O-glycan biosynthesis pathway may contribute to the intestinal barrier function which is impaired by influenza infection. Several pathways were downregulated by QFYD including inflammatory mediator regulation of TRP channels, MAPK signaling pathway, TNFα signaling pathway, and JAK-STAT signaling pathway. The downregulation of the inflammatory pathway indicates that QFYD may play an anti-inflammatory role in combating influenza infection. (Figures 5E–H).

### The Effect of Qingfeiyin Decoction on the Cecum Content

**FIGURE 6:**
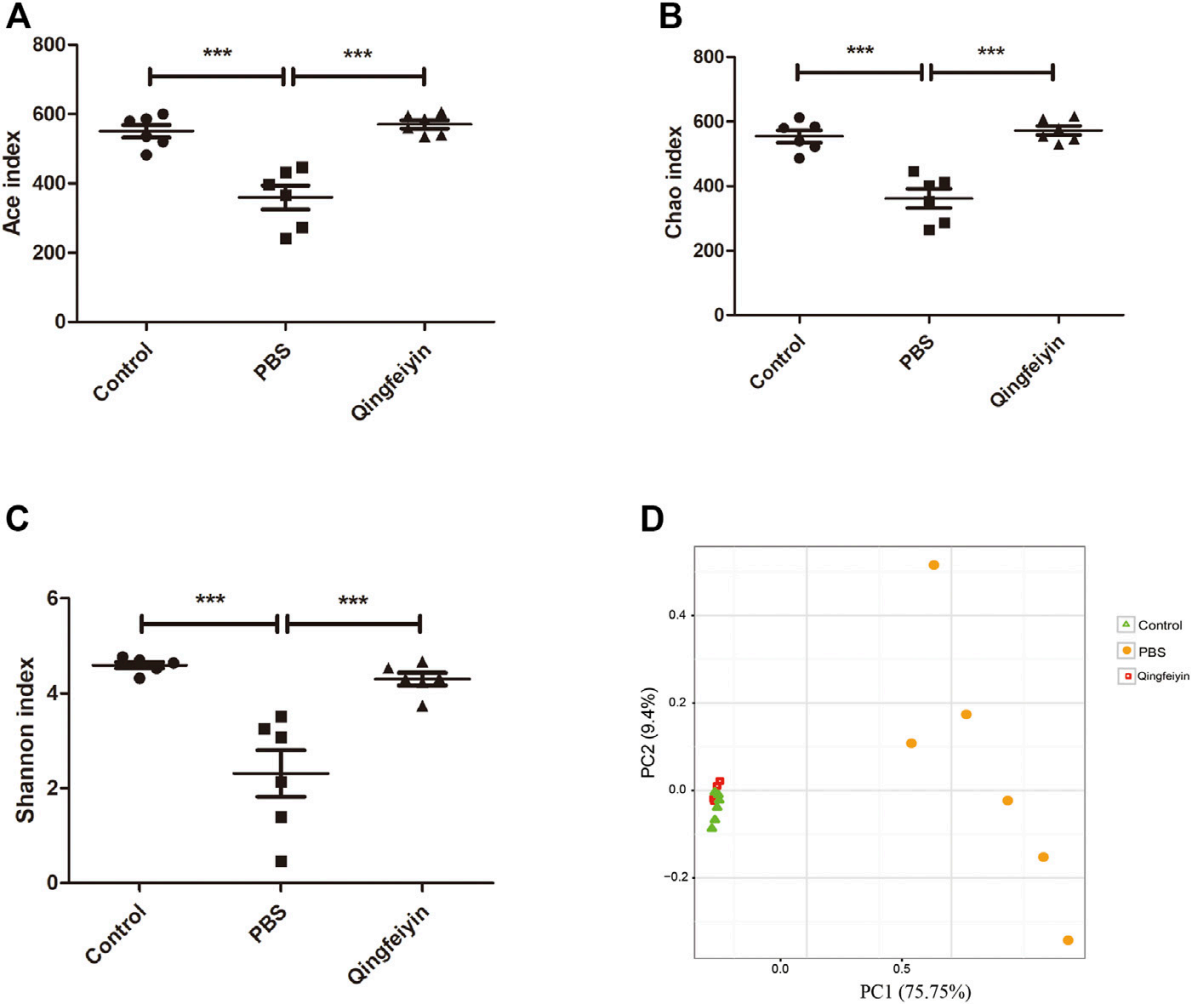
α and β diversity of cecum content. **(A–C)**, represents Ace, Chao, and Shannon indexes, respectively. **(D)** represents the plot of principal component analysis (PCA). The data **(A–C)** were analyzed using one-way ANOVA followed by Tukey’s test (*p* < 0.05). The data of D were analyzed using nonparametric MANOVA based on Adonis. ***p < 0.001.

It is believed that gut microbiota plays a key role in interacting with TCM. Therefore, 16 S rRNA was performed to observe the effect of QFYD on gut microbiota. The data of Ace, Chao, and Shannon indexes showed that the H1N1 virus remarkably increased the indexes, and QFYD treatment restored the indexes to the normal level so that there was no significant difference in α diversity between the control and QFYD group (Figures 6A–C). Principal component analysis (PCA) in Figure 6D showed that the control and QFYD groups almost completely clustered together, while the PBS group appeared distinct from the other two groups and had greater dispersion.

**FIGURE 7:**
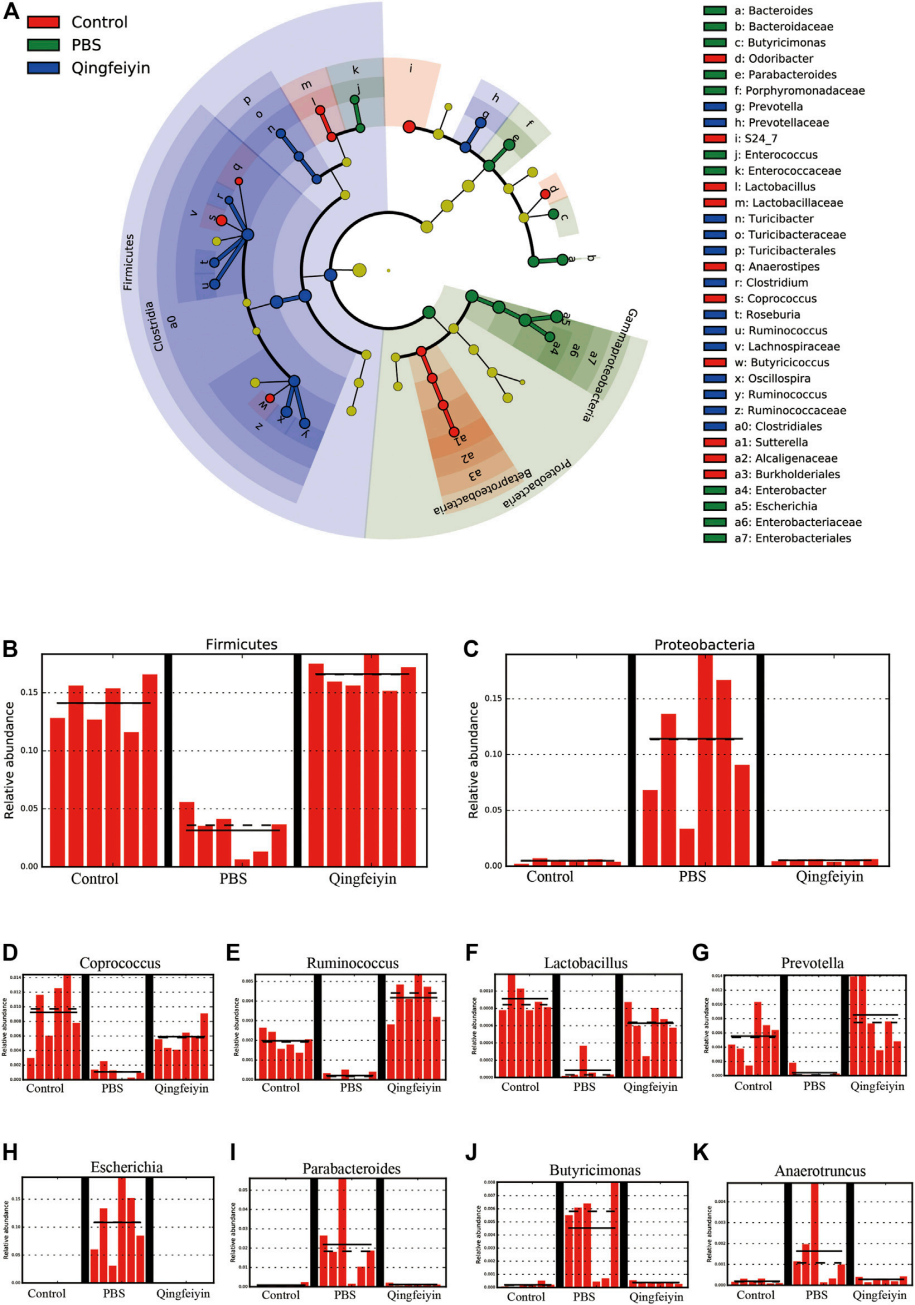
LEfSe analysis of cecum content. **(A)**, cladogram graph, **(B,C)** are histogram plots of the phylum of Firmicutes and Proteobacteria. **(D–K)** represent the genus of *Coprococcus*, *Ruminococcus*, *Lactobacillus*, *Prevotella*, *Escherichia*, *Parabacteroides*, *Butyricimonas*, and *Anacrotruncus,* respectively.

Stack diagram of cecum content showed that H1N1 infection increased the relative abundance of the Proteobacteria phylum, and decreased the relative abundance of the Firmicutes phylum. However, QFYD treatment reversed this trend making it similar to the level seen in the control group (Figures 7A–C; Supplementary Figure S1A). At the level of family, the H1N1 virus decreased the relative abundance of S24-7 and Lachnospiraceae and increased that of Enterobacteriaceae (Supplementary Figure S1B).

To further distinguish the differences of OTUs, a Lefse analysis was performed. The H1N1 virus decreased the relative abundance of the genera of *Coprococcu, Ruminococcus, Lactobacillus, and Prevotella*, while increasing the level of genera of *Escherichia, Parabacteroides, Butyricimonas,* and *Anacrotruncus*. QFYD treatment restored the composition of the cecum microbiota to a level comparable to that seen in the control group (Figures 7D–K).

### Spearman’s Correlation of Body Weight, Viral Load, the Differential OTUs, and the Differential Genes

**FIGURE 8:**
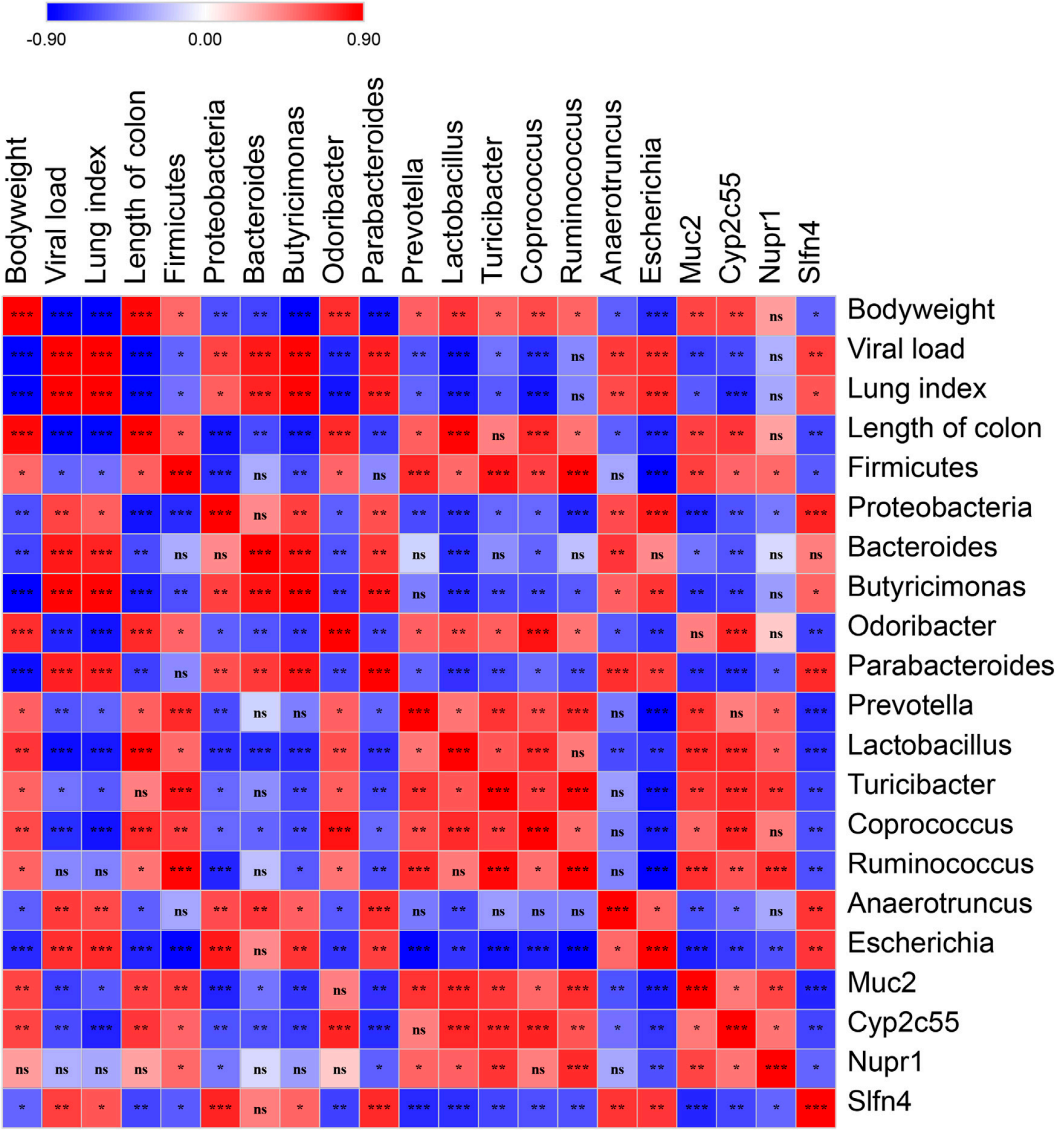
Spearman’s correlation graph of bodyweight, viral load, the differential OTUs, and the differential genes. The R values are denoted with graduated colors, and the red and blue grids indicated positive and negative correlations, respectively. (“ns” indicates non-significance; *p < 0.05, **p < 0.01, ***p < 0.001).

To validate the correlation of disease indices with gut microbiota and colonic gene expression, Spearman’s correlation analysis was performed based on the Spearman’s rank correlation coefficient. The data showed that viral load was significantly and positively correlated with lung index, the relative abundance of the Proteobacteria phylum, the genera of *Bacteroides, Butyricimonas, Parabacteroides, Anaerotruncus,* and *Escherichia*, as well as the expression of *Slfn4*, and negatively correlated with the length of the colon, the relative abundance of the Firmicutes phylum, the genera of *Odoribacter, Prevotella, Lactobacillus, Turicibacter,* and *Coprococcus,* as well as the expression of *Muc2* and *Cyp2c55* (Figure 8).

## Discussion

Qingfeiyin decoction is a traditional Chinese medicine, and it has been formulated and used clinically to treat influenza by experienced clinicians at Beijing University of Chinese Medicine Dongzhimen Hospital. It has been shown to be as effective as oseltamivir in treating influenza in a mouse model study, which suggests that Qingfeiyin may be used as an alternative therapy for influenza. There were 16 major chemical components identified that had higher than 1,000,000 in the intensity of QFYD, and the flavonoids including baicalein, wogonoside, chrysosplenetin B, chrysin, liquiritin, glabridinwas, and sanggenon H were the most abundant components. Previous studies have indicated that flavonoids can inhibit influenza and attenuate H1N1-induced acute lung injury (Bang et al., 2018; Ling et al., 2020; Yu et al., 2020), probably *via* inhibiting TLR signaling (Ling et al., 2020), suppressing NOX4/NF-κB/MLCK pathway (Yu et al., 2020), and inhibiting influenza H1N1 virus neuraminidase (Bang et al., 2018). A recent study reported that flavonoids could prevent SARS-CoV-2 infection by activating the transcription factor Nrf2 (Mendonca and Soliman, 2020). Flavonoids may also work as an angiotensin-converting enzyme 2 inhibitors for anti-SARS-CoV-2 (Muchtaridi et al., 2020). Forsythoside I, the second most abundant chemical component in QFYD, was also shown to inhibit influenza virus replication in mice (Deng et al., 2016; Law et al., 2017). Together, these studies suggest that multicomponents of QFYD may contribute to its anti-influenza effect *via* complex mechanisms.

The ancient TCM theory of “the lung and the large intestine are interior-exterior” was in accordance with what is demonstrated in the study “lung inflammation originating in the gut” by modern research (Huang et al., 2018; Mjösberg and Rao, 2018). In our study, we found that the colon length of mice infected with the H1N1 virus was significantly shorter than that of normal mice and was partly restored by QFYD treatment to a level similar to that of the control group. Wang *et al*. reported that respiratory influenza virus infection also induced intestinal immune injury and shortened the length of the colon (Wang et al., 2014), which is consistent with our findings. To explore the underlying mechanism, the transcriptome of colon tissue was performed. The data indicate that the influenza virus markedly decreased the expression of *Muc2*, while QFYD restored the expression of *Muc2*. Muc2 mucin plays a key role in protecting the gut barrier, maintaining microbiota homeostasis (Liu et al., 2020), and preventing colitis (Van der Sluis et al., 2006; Leon-Coria et al., 2021). GSEA also showed that the signaling pathway of mucin type o-glycan biosynthesis was upregulated by QFYD. Increased *Muc2* expression may be attributed to the upregulation of mucin type o-glycan biosynthesis. QFYD also downregulated inflammatory signaling pathways, including MAPK, TNFα, and JAK-STAT signaling pathways, which are important inflammatory signaling pathways and targets in the progression of influenza (Billmeier et al., 2016; Ma et al., 2020; Salas et al., 2020). The downregulation of inflammatory signaling pathways caused by QFYD is in favor of the maintenance of intestinal homeostasis.

Gut-lung axis theory indicates that the composition and function of gut microbiota are closely related to the immune responses and disease development in the lung (Marsland et al., 2015; Budden et al., 2017; Dang and Marsland, 2019). Reported that influenza infection altered the composition of small intestinal microbiota Wang et al. (2014), we explored the microbiota in the content of cecum and the major interacted site between herbs and microbiota. Wang’s study indicated that the copies of the *Lactococcus* were significantly lower, and Enterobacteriaceae was markedly higher in the small intestinal content of mice infected with influenza virus (Wang et al., 2014), which was consistent with our results in the content of cecum. Spearman’s correlation analysis showed viral loading has a strong negative correlation with the relative abundance of the genera of *Odoribacter, Prevotella, Lactobacillus*, and *Coprococcus*, as well as a strong positive correlation with the genera of *Bacteroides, Butyricimonas, Parabacteroides, Anaerotruncus*, and *Escherichia*. Previous studies showed that in influenza virus-infected mice, the relative abundance of genera of *Parabacteroidetes, Odoribacter, Coprococcus, Roseburia, Defluvittalea, Dorea, Ruminococcus*, and *Gemmiger* was higher, and in contrast, lower relative abundances were observed for the *Clostridium, Dehalobacterium*, and *Lactobacillus* genera (Sencio et al., 2020). In human studies, the dominated genera were *Enterococcus, Prevotella, Finegoldia,* and *Peptoniphilus* in H1N1 infected patients (Gu et al., 2020), and the genera of *Corynebacterium, Enterococcus, Rothia, Megasphaera*, and *Campylobacter* were significantly enriched, while *Eubacterium* was depleted in COVID-19 patients (Cao et al., 2021). In patients critically infected by COVID-19, there were severe dysbiosis in gut microbiota and enrichment of potential pathogens, particularly *Enterococcus* (Gaibani et al., 2021). The differences in the levels of higher or lower genera in previous studies may be due to the different types of influenza virus or different conditions of the hosts. However, all studies shared similarities that influenza caused a higher abundance of *Enterococcus* and a lower level of *Lactobacillus*. Therefore, we hypothesize that increasing the level of *Lactobacillus* by direct oral administration of probiotic *Lactobacillus* or indirectly by administration of prebiotics to promote the growth of *Lactobacillus*, may be effective for the treatment of influenza.

In summary, in this study, we found that QFYD decreased viral loading and increased the length of the colon caused by the H1N1 virus, probably *via* modulation of gut microbiota and downregulation of inflammatory pathways in the colon. We need to further observe the effect of multiple doses of QFYD on influenza-infected mice in future study. QFYD has been used clinically at Beijing University of Chinese Medicine Dongzhimen Hospital for decades. Since the mechanisms learned from animal models may not be the same as what happens in patients, in future studies, we will use fecal and blood samples collected from influenza patients treated with QFYD to validate and further explore the mechanisms for QFYD’s anti-influenza effect.

## Data Availability

The datasets presented in this study can be found in online repositories. The names of the repository/repositories and accession number(s) can be found at: https://www.ncbi.nlm.nih.gov/bioproject/, PRJNA765210; PRJNA765456.
