# Supplementary figures and images for: Qingfeiyin Decoction Inhibits H1N1 Virus Infection via Modulation of Gut Microbiota and Inflammatory Pathways in a Murine Model
Source: Front Pharmacol. 2022 May 23;13:874068. doi: 10.3389/fphar.2022.874068 (PMC9170074; doi:10.3389/fphar.2022.874068)

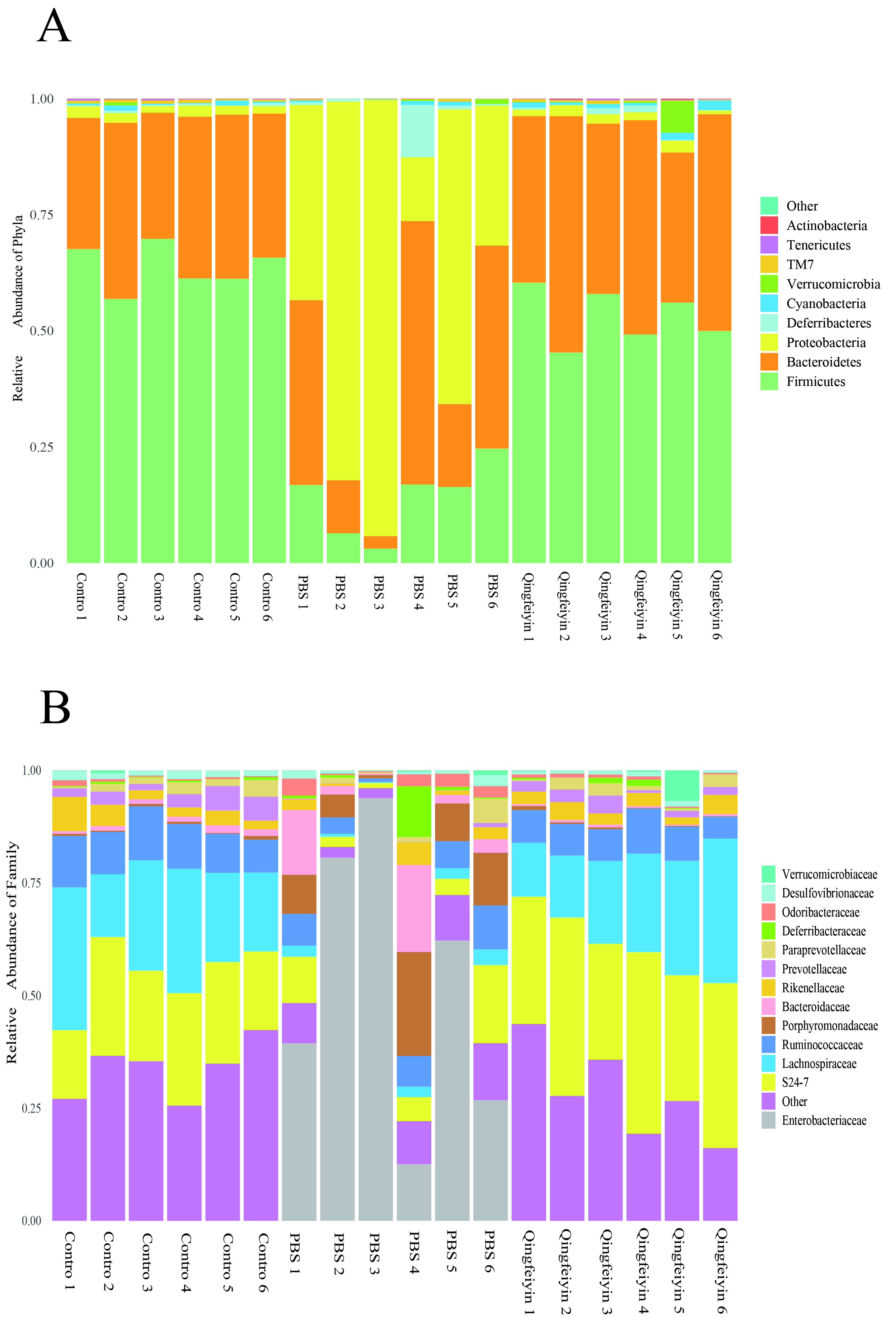

Supplement: Supplementary file 1 [file Image1.JPEG]
